# Insights into Metabolic Engineering of Bioactive Molecules in *Tetrastigma hemsleyanum* Diels & Gilg: A Traditional Medicinal Herb

**DOI:** 10.2174/0113892029251472230921053135

**Published:** 2023-10-27

**Authors:** T.P. Ajeesh Krishna, T. Maharajan, T.P. Adarsh Krishna, S. Antony Ceasar

**Affiliations:** 1 Division of Plant Molecular Biology and Biotechnology, Department of Biosciences, Rajagiri College of Social Sciences, Kochi, 683104, Kerala, India;; 2 Research & Development Division, Sreedhareeyam Farmherbs India Pvt. Ltd, Ernakulam, 686-662, Kerala, India

**Keywords:** Drug discovery, genome editing, flavanoids, metabolic engineering, *Tetrastigma hemsleyanum*, bioactive molecules

## Abstract

Plants are a vital source of bioactive molecules for various drug development processes. *Tetrastigma hemsleyanum* is one of the endangered medicinal plant species well known to the world due to its wide range of therapeutic effects. Many bioactive molecules have been identified from this plant, including many classes of secondary metabolites such as flavonoids, phenols, terpenoids, steroids, alkaloids, *etc*. Due to its slow growth, it usually takes 3-5 years to meet commercial medicinal materials for this plant. Also, *T. hemsleyanum* contains low amounts of specific bioactive compounds, which are challenging to isolate easily. Currently, scientists are attempting to increase bioactive molecules' production from medicinal plants in different ways or to synthesize them chemically. The genomic tools helped to understand medicinal plants' genome organization and led to manipulating genes responsible for various biosynthesis pathways. Metabolic engineering has made it possible to enhance the production of secondary metabolites by introducing manipulated biosynthetic pathways to attain high levels of desirable bioactive molecules. Metabolic engineering is a promising approach for improving the production of secondary metabolites over a short time period. In this review, we have highlighted the scope of various biotechnological approaches for metabolic engineering to enhance the production of secondary metabolites for pharmaceutical applications in *T. hemsleyanum*. Also, we summarized the progress made in metabolic engineering for bioactive molecule enhancement in *T. hemsleyanum.* It may lead to reducing the destruction of the natural habitat of *T. hemsleyanum* and conserving them through the cost-effective production of bioactive molecules in the future.

## 1 INTRODUCTION

Naturally occurring molecules have high economic value and are essential ingredients in cosmetics, fragrances, food additives, agrochemicals, dyes, pharmaceuticals, *etc*. Plants are the primary source of active ingredients used as drugs for human health care purposes [1]. Many effective drug molecules are derived from plant origins [2]. It is safe for human health and has no side effects compared to synthetic drugs. *Tetrastigma hemsleyanum* Diels & Gilg is an important and endangered medicinal plant species in the Vitaceae family. The demand for *T. hemsleyanum* in the therapeutic market increases daily due to its high medicinal importance. Due to its naturally slow growth rate usually takes 3-5 years to meet commercial restorative materials requirements [3, 4]. The plant has been recognized to be highly effective for various human ailments. The medicinal values and bioactive compound properties of *T. hemsleyanum* have been reviewed by many researchers [4-6]. In particular, this plant has a lot of flavonoid molecules, which have a solid ability to cure many human diseases. The bioactive constituents of *T. hemsleyanum* provide an opportunity to develop new drugs for emerging infectious diseases. The low availability of raw materials and active bioactive molecules from plants increases their demand and hampers their usage for human health care. Therefore, there is an urgent need for alternative ways for rapid and large-scale production of bioactive molecules from *T. hemsleyanum*. It helps reduce the demand for bioactive molecules from *T. hemsleyanum* and may lead to the development of novel active drug ingredients for human health care.

Nowadays, scientists are trying to enhance the production of bioactive molecules from medicinal plants or synthesize them in different fields. These two research areas could be beneficial for enhancing/developing bioactive molecules. Biotechnologists are trying to improve the production of bioactive molecules from medicinal plants using various approaches. In past decades, plant cell culture methods were used for secondary metabolite production [7, 8]. State-of-the-art genome sequencing approaches are available now, and they help provide insight into medicinal plants' genome organization [9]. Therefore, functional genomics/transgenic approaches provide the opportunity to understand genes and their role in the involvement of biosynthesis pathways associated with secondary metabolites [10]. It helps manipulate the natural biosynthesis pathway of the production of bioactive molecules and may increase their production.

Furthermore, the advanced genome editing approach helps to target the gene of interest in the biosynthetic pathway of the active molecules [11], which allows for the alteration of the production of bioactive molecules. Therefore, advances in metabolic engineering have enabled enhanced production of desired bioactive molecules and introduced novel biosynthetic pathways to desired bioactive molecules. Another concern is that some active molecules yield very low in medicinal plants, and isolating them from natural sources is challenging. However, organic chemists also develop new synthetic routes for producing bioactive molecules and their analogs [12]. However, the synthesis of natural molecules has some limitations. Bioactive molecules with simple structures can be easily synthesized, but a complex structure with multiple chiral centers makes it challenging to synthesize them chemically [13, 14]. Also, synthetic molecules have some side effects due to their chemical toxicity. Therefore, excessive use of synthetic drugs can seriously affect human health. In this review, we have highlighted the scope of various biotechnological approaches for metabolic engineering to improve the production of flavonoid molecules from *T. hemsleyanum.* This review is beneficial for plant physiologists to understand the current progress in enhancing bioactive molecules' production from *T. hemsleyanum*. It may also lead to reducing the usage of endangered plant species like *T. hemsleyanum* and conserving them in the future.

## 2 MEDICINAL IMPORTANCE OF *T. HEMSLEYANUM*

*T. hemsleyanum* is the most important folklore medicinal plant species in China. It has been recorded based on the usage of this plant for various ailments in humans. Different plant parts of *T. hemsleyanum* are used for internal or external applications for various health benefits. For example, the leaves are consumed internally as a functional tea or dietary supplement that helps to improve the human immune system [15]. Similarly, root tubers are commonly used individually or in combination with other herbal medicines to treat lung-related ailments like pneumonia and asthma [16-18]. Also, root tubers are externally applied to cure adverse joint flexion and extension [4]. It has been frequently used to treat hepatitis, infantile high fever, gastritis, cervicitis, lymphatic tuberculosis, menstrual disorders, pharynx pain, septicemia, viral meningitis, snake bites, *etc*. [4, 5]. Therefore, this plant species is known worldwide as a source of phytotherapeutics. The whole plant has a rich source of bioactive molecules. Due to their remarkable bioactivities, *T. hemsleyanum* is widely used in Chinese herbal medicine. So far, around 142 biochemical constituents have been identified from *T. hemsleyanum*, including many compounds such as flavonoids, phenols, terpenoids, steroids, alkaloids, *etc* [4]. The abundance of this class of bioactive compounds provides their importance in drug discovery.

**Fig. (1):**
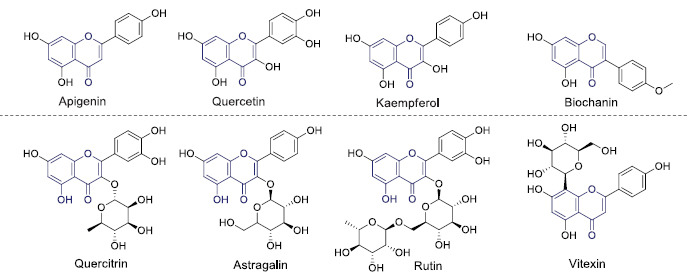
Selected flavonoids and glycosylated flavonoids from *T. hemsleyanum.* These molecules have a wide range of biological activities. (Created with BioRender.com)

Recent progress in phytochemical screening revealed that flavonoids are the primary bioactive molecules in *T. hemsleyanums* [19, 20]. Therefore, researchers have focused on identifying and characterizing flavonoid class molecules from *T. hemsleyanum*. Many researchers have isolated large amounts of flavonoid content in leaves, aerial parts, and root tubers of *T. hemsleyanum* [19-21]. It shows a wide range of biological activity, especially anti-cancer activity. For example, Feng *et al.* [21] analyzed that the antitumor activity of total flavonoids from *T. hemsleyanum* is associated with inhibiting regulatory T-cells in mice. This study revealed that the total flavonoids significantly inhibited tumor growth in mice (C57BL/6) inoculated with Lewis lung carcinoma (LLC) and suppressed regulatory T-cell development in tumor-bearing mice. The total flavonoids significantly decreased the serum levels of transforming growth factor β, prostaglandin E2, and cyclooxygenase 2 in tumor-bearing mice, which may be responsible for inhibiting regulatory T-cells [21]. Similarly, many researchers have scientifically proven the anti-cancer activity of flavonoid molecules from *T. hemsleyanum* [19, 22-26]. Progress in advanced techniques in phytochemistry, many bioactive flavonoid molecules have been detected from *T. hemsleyanum* so far (Fig. **1**). Several reports are available on the biological activities of these flavonoid classes of molecules in *T. hemsleyanum*. Also, many clinical and animal model studies show these plant compounds possess many biological properties. Therefore, it is urgently needed to enhance the production of secondary metabolites, especially flavonoid molecules from *T. hemsleyanum,* and it could help more clinical studies for emerging infectious diseases.

## 3 RECENT PROGRESS FOR METABOLIC ENGINEERING IN *T. HEMSLEYANUM*

Active secondary metabolites can be isolated from naturally growing plants. But, it takes several years for plants to develop to the point where they can produce the desired metabolites. The *T. hemsleyanum* is a perennial plant species. Its tubers are slow-growing in nature, taking 3-5 years of growth to get commercial medicinal materials. Also, their commercial production is restricted due to regional and environmental constraints. Sunlight, temperature, humidity, precipitation, soil fertility, and salinity are environmental variables that can influence the metabolite pathways, changing the phytochemical profiles and production of bioactive molecules [27]. Recently, Shi *et al.* [28] revealed that seasonal variation influences flavonoid biosynthesis path and content in *T. hemsleyanum*. Therefore, scientists are looking for alternative approaches to increase the production of active secondary metabolites from these plants. Recent progress in characterizing the biosynthesis pathway of metabolites has provided the opportunity to identify the genes involved in the biosynthesis pathway. Therefore, manipulating such genes provides promising approaches for improving productivity in a plant cell, tissue, and organ culture (PCTOC). Various metabolic engineering approaches are available to enhance the production of desirable bioactive molecules from medicinal plants. It includes transgenic, genomic, and genome editing approaches. The past decade has seen impressive progress in plant metabolic engineering using these approaches.

### 3.1 Pant Cell, Tissue, and Organ Culture

**Fig. (2):**
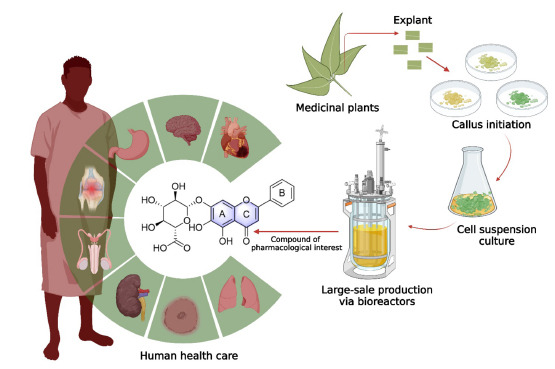
Plant cell, tissue, and organ culture (PCTOC) approach for producing secondary metabolites. The optimum culture media helps to accumulate desirable secondary metabolites within a short period. The desirable secondary metabolites have high therapeutic values it helps human health care.

The PCTOC strengthens metabolic engineering in different ways. In the past, the PCTOC techniques also helped to produce secondary metabolites efficiently within a short duration for commercial application [29, 30]. This approach could provide a continuous supply of uniform quality, desirable, natural components. In recent decades, significant developments have been made in the PCTOC. Recently, the focus has shifted to improving the culture conditions for metabolite production through screening and selection of high-producing cell lines, media optimization, elicitation, precursor feeding, and two-phase co-culture among PCTOC approaches. These efforts have been made with the possibility to scale up the production of secondary metabolites, meet the pharmaceutical industry's demand, and conserve natural sources of secondary metabolites. Subsequently, suspension culture systems emerged as an immediate method for producing secondary metabolites [31]. In suspension cultures, plant cells, tissues, or organs are inoculated into a large liquid medium container with suitable cell growth stimulants. The production of secondary metabolites is based on the biosynthetic totipotency of the plant cell. The production of secondary metabolites in the cell suspension culture occurs through the plant cell's biosynthetic totipotency. Also, it has helped produce more desirable bioactive molecules from medicinal plants. So far, many researchers have established an efficient PCTOC protocol for improving the production of secondary metabolites in various medicinal plants [32-37]. For example, the anticancer compound podophyllotoxin is produced in *Linum album* through *in vitro* callus culture [38]. Also, various secondary metabolites, such as camptothecin [39], hypericin, hyperforin [40], taxol [41], apigenin [42], paclitaxel [43], cephalotaxine [44], reserpine, ajmalicine [45, 46], atropine [47], *etc*. have been successfully produced through *in vitro* cell suspension culture. Therefore, this approach could be suitable for commercially producing bioactive molecules from various medicinal plants (Fig. **2**).

**Fig. (3):**
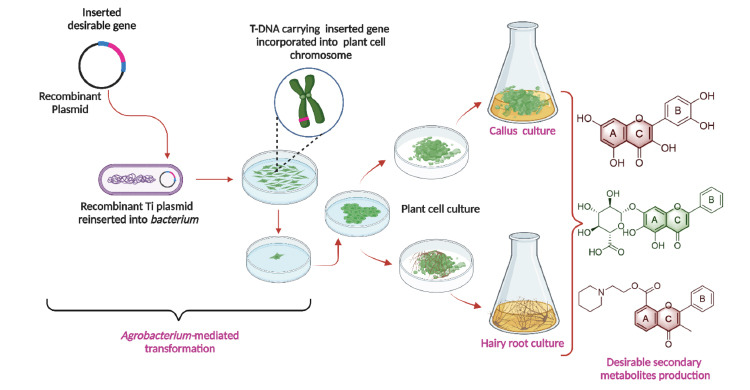
Transgenic approaches for metabolic engineering in plant cell culture. The *Agrobacterium*-mediate transformation is the most common way to introduce heterologous DNA (desirable genes) in plants. It is one of the efficient methods for metabolic engineering approaches widely used by plant biotechnologists. It needs efficient tissue culture and transformation protocols.

We know that flavonoid molecules from *T. hemsleyanum* are in high demand due to their potential medicinal value. However, little effort has been made to enhance the production of secondary metabolites from *T. hemsleyanum*, especially flavonoids, through PCTOC. Only Peng's research group has developed an efficient protocol for the *T. hemsleyanum* callus culture in a liquid medium [48-51]. The optimum callus growth has been observed under the MS medium supplemented with 3 mg/L BA, 2 mg/L naphthaleneacetic acid (NAA), and 2 g/L peptone. Also, the combination of B5+ BA 4 mg/L + NAA 2 mg/L + Phenylalanine 40 mg/L culture medium and photoperiods of 24 h light showed the best flavonoid accumulation in callus culture. These studies revealed that cultured callus's total flavonoid content (28.4 ± 3.9 mg/g in dry weight) was significantly higher than that of raw plant leaves of *T. hemsleyanum* [48]. It shows that the PCTOC is a promising approach for improving the secondary metabolites from *T. hemsleyanum*. Furthermore, the same research group screened the effect of metal [silver (Ag^+^), copper (Cu^2+^), cadmium (Cd^2+^), cerium (Ce^3+^), calcium (Ca^2+^), lanthanum (La^2+^), neodymium (Nd^3+^)] ions on the increase in callus of *T. hemsleyanum* suspension cell cultures to improve the production of flavonoid contents [51, 52]. It revealed that the metal ions help to enhance the production of biomass of callus and flavonoid contents in suspension culture. For example, under-treatment of 100 μM Ce^3+^and Nd^3+^over 25-day culture periods significantly increased the total callus biomass by 1.92- and 1.74-fold and the total flavonoid contents by 1.45- and 1.49-fold, compared with control. No further reports are available on metabolic production in *T. hemsleyanum* through PCTOC. Combining transgenic approaches with PCTOC provides the opportunity to produce better secondary metabolites from plants (Fig. **3**). Therefore, researchers need to focus on developing an efficient PCTOC protocol for producing desirable active molecules from *T. hemsleyanum*. It could help the commercial production of desirable bioactive molecules and reduce the destruction of the natural habitat of *T. hemsleyanum*. It could allow for conserving these plants in the future.

### 3.2 Transgenic Approaches

The genetic engineering of metabolic pathways in plants requires manipulating one or more genes at their genome level. The manipulation of existing metabolic pathways by overexpressing desirable genes involved in their biosynthesis is a key technique that enables the overproduction of valuable plant secondary metabolites. The transgenic approaches allow the insertion of desirable genes into plant genomes [53]. Therefore, it provides enormous possibilities for plant genetic improvements [54]. This approach requires efficient plant tissue culture and genetic transformation protocols. In the past decades, an *Agrobacterium tumefaciens*-mediated transformation system has been developed by which several genes related to the biosynthesis pathway have been successfully transferred into plants (Fig. **3**). It has helped to enhance the production of desirable secondary metabolites. For example, the overexpression of *3-hydroxy-3-methylglutaryl CoA synthase 1* (*HMGS1*) has improved the total content of terpenoids (lanosterol, dehydroabietic acid, and phytol) in the transgenic *Populus* plants [55]. Unfortunately, *Agrobacterium tumefaciens*-mediated transformation system has not yet been successfully developed in *T. hemsleyanum*. But, many researchers have established an efficient tissue culture protocol using various explants [56-58], which could help to develop an *Agrobacterium tumefaciens*-mediated transformation system easily in *T. hemsleyanum.* Therefore, plant biotechnologists must focus on developing an efficient *Agrobacterium tumefaciens*-mediated transformation system in *T. hemsleyanum* due to its medicinal importance. It may help to produce the desirable secondary metabolites from *T. hemsleyanum.* Furthermore, the transgenic hairy root culture is a promising approach to synthesize secondary metabolites. Previous research has demonstrated that the transgenic hairy root has a higher capacity for the biosynthesis of secondary metabolites than the non-transgenic roots of various plants. Therefore, *Agrobacterium rhizogenes*-based transformation is another potential system for enhancing the production of secondary metabolites. Du *et al.* [3] developed hairy roots through infection of the *T. hemsleyanum* leaf explant using *Agrobacterium rhizogenes* strain K599. This study revealed that the combination of MS + IBA 1.0 mg/L + kinetin 0.5 or 1.0 mg/L was the best medium for the subculture of hairy roots. In suspension culture, the rapid growth phase took place over 15-28 days, and the contents of kaempferol in all hairy root cultures have been significantly higher than those in root tubers, fine root, stems, and leaves [3].

Overall, the *Agrobacterium*-mediated transformation helps to insert genes to alter the metabolic pathways to increase the production of secondary metabolites. Also, it helps to characterize the gene function through their overexpression in planta. Therefore, researchers must actively identify the valuable genes and transcription factors (TF) involved in metabolic pathways in *T. hemsleyanum*.

### 3.3 Genomic Approaches

The genomic approach helps to trace the key genes controlling metabolic pathways in plants. Nowadays, the next-genome sequence (NGS) platform allows whole genome and transcriptome analysis to mine the candidate genes and TFs related to the biosynthesis of secondary metabolites. The genome sequence technology provided the opportunity to understand the genome organization of many plant species, including *Rosa roxburghii* [59], *Acer truncatum* [60], *Hypericum perforatum* [61], *Senna tora* [62], *Vernicia fordii* [63], *Platycodon grandiflorus* [64], *Ocimum tenuiflorum* [65], *Camptotheca acuminata* [66], *Magnolia biondii* [67], *etc*. For example, Dong *et al.* [67] have done the genome assembly of *Magnolia biondii via* three different genome sequence platforms. Further analysis revealed that some specific genes are associated with the biosynthesis of alkaloids, ubiquinone, terpenoids, quinones, phenylpropanoids, and other secondary metabolites. It provided an understanding of their metabolite's biosynthesis and regulation of bioactive molecules. Therefore, overexpression of these specific genes helps to improve the desirable secondary metabolites. In this context, the lack of a whole genome sequence of *T. hemsleyanum* hampers the possibility of doing metabolic engineering. Therefore, due to their medicinal importance, the plant research group must focus on whole genome sequencing in *T. hemsleyanum.* It may provide a breakthrough for enhancing bioactive molecule production from *T. hemsleyanum.*

**Fig. (4):**
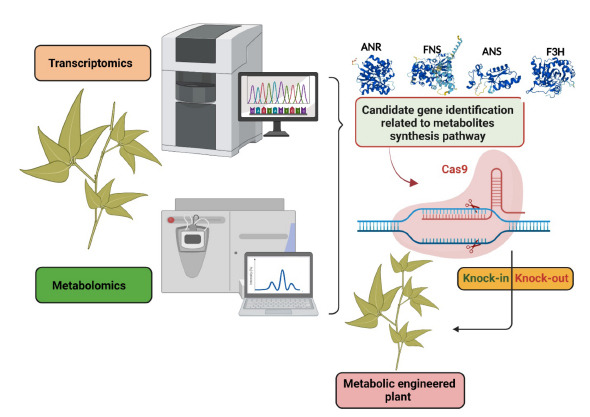
Metabolic engineering through CRISPR/Cas9 genome editing system. Metabolomics and transcriptomics analysis help to identify the candidate genes related to metabolic pathways. The CRISPR/Cas9 genome-editing system allows to alteration of metabolic production through the knock-in or knock-out of desirable genes.

However, little information is available on the transcriptome sequencing data of T. *hemsleyanum* related to metabolomics. Like whole genome sequencing, transcriptome sequencing allows mining the candidate gene related to the biosynthesis of secondary metabolites. Recently, Bai *et al.* [68] investigated the flavonoid metabolism of *T. hemsleyanum via* metabolome analysis and transcriptome sequencing. The metabolomic analysis shows that flavonoid content varied between the leaves and root tubers of *T. hemsleyanum*. Further, the transcriptome analysis revealed that many differentially expressed genes (DEGs) like *chalcone isomerase* (*CHI*) and *UDP-glycose flavonoid glycosyltransferase* (*UFGT*) are playing a critical role in flavonoid metabolism in the leaves and root tubers of *T. hemsleyanum* [68]. Also, many other genes involved in various metabolite pathways were identified *via* transcriptome analysis in *T. hemsleyanum* [68]. Similarly, many researchers have analyzed the transcriptome sequencing related to metabolomics in *T. hemsleyanum* [69-73] and identified DEGs related to metabolite synthesis. Therefore, the DEGs related to secondary metabolites synthesis could be helpful for metabolic engineering *via* transgenic or other genome-editing approaches (Figs. **3** and **4**). Therefore, researchers need to focus on this area of research.

### 3.4 Genome-editing Approaches

A DNA sequence can be altered using the site-specific nucleases by removal, insertion, or mutation of basses at the targeted locus by genome editing tools [74]. Therefore, genome-editing tools help to target any gene of interest precisely and improve plant traits [75, 76]. In the past decades, genome editing tools like zinc-finger nucleases (ZFNs) and transcription activator-like effector nucleases (TALENs) have been popularized for transcriptional-level genome manipulation [11, 77]. These genome-editing tools enable genetic alterations by inducing DNA double-strand breaks (DSBs) that stimulate error-prone nonhomologous end joining (NHEJ) or homology-directed repair (HDR) at specific genomic locations [78]. But, ZFNs and TALENs tools demand laborious efforts for cloning and protein construction to make DSBs [79, 80]. Clustered, regularly interspaced short palindromic repeat (CRISPR)/CRISPR-associated protein 9 (Cas9) has emerged as a user-friendly tool for genome engineering [81]. The CRISPR/Cas9 system is an efficient, robust, and selective site‐directed mutagenesis strategy for RNA-guided genome editing. The researchers have utilized the CRISPR/Cas9 system for various applications, including metabolic engineering. It helps to enhance/reduce the production of desirable secondary metabolites in medicinal plant species. The CRISPR/Cas9 system has been successfully employed in medicinal plants such as *Salvia miltiorrhiza* [82-84], *Atropa belladonna* [85, 86], *Dioscorea zingiberensis* [87], *Cichorium intybus* [88] *Medicago truncatula* [89] *Opium poppy* [90], *Symphytum officinale* [91], *etc*. for metabolic engineering. For example, CRISPR/Cas9-mediated mutagenesis of the *hyoscyamine 6β-hydroxylase (H6H)* gene completely disrupted the conversion from hyoscyamine to anisodamine and scopolamine in *Atropa belladonna* [86]. As a result, the production of hyoscyamine increased in the mutant lines of *Atropa belladonna* [86]. The *homospermidine synthase* (*HSS*) gene is involved in the biosynthesis of pyrrolizidine [92, 93]. The pyrrolizidine is a toxic alkaloid compound found in many medicinal plant species that causes hepatic failure [94-96]. Knock-out of the *HSS* gene using the CRISPR/Cas9 system showed the reduction of pyrrolizidine alkaloid level in the mutated lines of *Symphytum officinale* [82]. Furthermore, the CRISPR-Cas9 system was successfully used to alter the biosynthetic pathway of alkaloids in *Opium poppy* [90], carotenoid in *Ipomoea nil* [97], flavonoids in *Fagopyrum tataricum* and the phenolic metabolism in *Salvia miltiorrhiza* [82] *etc* (Table **1**). Therefore, CRISPR/Cas genome-editing systems provide a vast scope in medicinal plant metabolic engineering. So far, no research has been undertaken on genome editing in *T. hemsleyanum* using the CRISPR/Cas9 system. It is due to the lack of genomic information, efficient tissue culture, and genetic transformation protocols. In transcriptome analysis, many DEGs related to various metabolic pathways have been identified in *T. hemsleyanum* (Table **2**). Therefore, the knock-in or knockout of desirable genes through the CRISPR-Cas9 system could help alter the metabolic pathway and enhance the production of desirable secondary metabolites in *T. hemsleyanum* (Fig. **4**). It may give a breakthrough to the pharmaceutical industry for the efficient production of metabolites.

## CONCLUSION

*T. hemsleyanum* is one of the endangered medicinal plant species with a wide range of therapeutic values. Therefore, researchers are focusing on this plant for new drug discovery. This plant's flavonoid class of molecules showed potent activity against newly emerging infectious diseases. Therefore, a massive quantity of these active molecules is needed for further clinical studies and drug development. In this context, the overuse of this plant species may cause it to become extinct from its natural habitat. Nowadays, metabolic engineering provides the opportunity to enhance the production of metabolites from medicinal plants. It could help reduce the overuse of *T. hemsleyanum*. Understanding the genes involved in the metabolic pathway is crucial for plants' metabolic engineering. The recent tools in genomics, functional genomics, and genome editing might contribute to better plant metabolic engineering. It helps identify and validate candidate genes involved in various pathways of secondary metabolites. But, only a little effort was made to improve secondary metabolites *via* metabolic engineering in *T. hemsleyanum*. This may be due to the lack of well-established genetic transformation protocols and genomic information on *T. hemsleyanum*. Therefore, researchers need to make efforts to improve the metabolic engineering approaches in this plant. Also, scientists need to improve the production of desirable bioactive molecules from *T*. *hemsleyanum* using various biotechnological approaches, especially CRISPR/Cas9 genome editing tools. It may help to enhance the desirable bioactive molecules from these plants and to do further clinical studies for new drug developments.

## Figures and Tables

**Table 1:** Applications of CRISPR/Cas genome-editing system to alter various secondary metabolites in medicinal plants. Details on medicinal plant, target gene, secondary metabolite altered, CRISPR editing type, method of construct delivery and results obtained were included.

**S. No.**	**Medicinal Plant Species**	**Target Gene**	**Secondary ** **Metabolites**	**Editing Type**	**Method of Delivery**	**Results**	**References**
1.	*Atropa belladonna*	*H6H*	Hyoscyamine	CRISPR/Cas9-mediated mutagenesis	*Agrobacterium*- mediated transformation	Increasing the production hyoscyamine	[86]
2.	*Banana cv. Grand Naine*	*LCYε*	β-carotene	CRISPR/Cas9-mediated mutagenesis	*Agrobacterium*- mediated transformation	Enhanced accumulation of β-carotene content	[98]
3.	*Cichorium intybus*	*GAS*	Sesquiterpene lactones	Knock-out by CRISPR/Cas9	PEG-mediated transfection	Inactivation of the sesquiterpene lactones biosynthesis pathway led to increase in phenolic compounds	[88]
4.	*Ipomoea nil*	*CCD4*	Carotenoid	Knock-out by CRISPR/Cas9	*Agrobacterium- mediated transformation*	Alter the carotenoid contents	[97]
5.	*Dioscorea zingiberensis*	*FPS*	Squalene	CRISPR/Cas9-mediated mutagenesis	*Agrobacterium*- mediated transformation	Reduced the content of squalene	[87]
6.	*Salvia miltiorrhiza*	*LAC*	Phenolic compounds	Knock-out by CRISPR/Cas9	*A. rhizogenes*- mediated transformation	Reduced the content of phenolic compounds such as RA and SAB	[82]
7.	*Salvia miltiorrhiza*	*RAS*	Phenolic compounds	Knock-out by CRISPR/Cas9	*A. rhizogenes*- mediated transformation	Reduced the content of phenolic compounds such as RA and LAB	[83]
8.	*Salvia miltiorrhiza*	*CPS1*	Tanshinones	CRISPR/Cas9-mediated mutagenesis	*A. rhizogenes*- mediated transformation	Reduced the content of tanshinones	[84]
9.	*Camellia sinensis*	*HB1 and NMT1*	Caffeine	CRISPR/Cas9-mediated mutagenesis	*Agrobacterium*- mediated transformation	Alter the expression level of *NMT1gene* related to caffeine biosynthesis pathway	[99]
10.	*Papaver somniferum*	*4′OMT2*	Benzylisoquinoline alkaloids	Knock-out by CRISPR/Cas9	*Agrobacterium*- mediated transformation	Reduced the content of benzylisoquinoline alkaloids (*e.g*., morphine, thebaine)	[90]
11.	*Symphytum officinale*	*HSS*	Pyrrolizidine alkaloids	Knock-out by CRISPR/Cas9	*A. rhizogenes*- mediated transformation	Reduced the content of pyrrolizidine alkaloids.	[91]
12.	*Fagopyrum tataricum*	*MYB45*	Flavonoids	Knock-out by CRISPR/Cas9	*A. rhizogenes*- mediated transformation	Increasing the production flavonoid compounds	[100]

**Abbreviations: *H6H,****hyoscyamine 6β-hydroxylase;****RAS,****rosmarinic acid synthase gene;****LAC,****laccase;****RA,****rosmarinic acid;****SAB,****salvianolic acid B;****LAB***, *lithospermic acid B****; HSS***, *homospermidine synthase;****4′OMT2***, *3-hydroxy-N-methylcoclaurine 4′–methyltransferase;****HB1***, *hemoglobin 1;****NMT1***, *N-methyltransferase;****GAS***, *germacrene A synthase;****FPS***, *farnesyl pyrophosphate synthase;****F3’H***, *flavonoid 3′-hydroxylase;****CCD4,****carotenoid cleavage dioxygenase 4;****LCYε***, *lycopene epsilon-cyclase;****CPS1,****diterpene synthase 1*.

**Table 2:** Details of differentially expressed genes (DEGs) related to the metabolites pathway in *T. hemsleyanum* through transcriptome analysis. Details on plant parts used, platform used for sequencing, stress condition, number of DEGs identified, some annotated DEGs and their functions were included.

**S. No.**	**Plant Parts Used for Transcriptome**	**Platform Used**	**Condition**	**No. of DEGs**	**Annotated DEGs**	**Functions**	**References**
1.	Plantlets	Illumina HiSeq	Cold stress	18,104	*PAL, F3’H, C4H, 4CL, CHS, F3H, ANR, FLS,* and *LAR*	Involved in flavonols biosynthesis	[69]
2.	Leaves and root tubers	Illumina HiSeq	-	67,345	*FNS, F3H, UFGT, PAL, CHS,* and *CHI*	Involved in flavonoid biosynthesis	[68]
3.	Cutting seedlings	Illumina HiSeq	Cold stress	7,883	*BG1, PAL, CCR, COMT, CHR,* and *CHS*	Involved in phenylpropanoid and flavonoid biosynthesis	[70]
4.	Leaves	Illumina HiSeq	-	-	*LAR, ANS, ANR,* and *DFR*	Involved in proanthocyanidin metabolism	[71]
5.	Leaves	Illumina HiSeq	-	55,373	*ADT, PAL, 4CL, C3H, CSE, HCT, CCoAOMT,* and *CHS*	Involved in phenylpropanoid and flavonoid biosynthesis	[72]
6.	Leaves	Illumina HiSeq	-	4211	*CHS, CHI, F3H, F3'H, F3'5'H, DFR, ANS,* and *UFGT*	Involved in anthocyanin biosynthesis	[73]

**Abbreviations: *4CL***, *4-coumaroyl: coenzyme A ligase;****ADT***, *arogenate/prephenate dehydratase;****ANR***, *anthocyanidin reductase;****ANS***, *anthocyanin synthase;****BG1***, *beta-glucosidase 1;****C3H***, *5-O-(4-coumaroyl)-D-quinate 30-monooxygenase;****C4H***, *cinnamate-4-hydroxylase;****CCoAOMT***, *caffeoyl-CoA O-methyltransferase;****CCR***, *cinnamoyl-CoA reductase;****CHI***, *chalcone isomerase;****CHR***, *chalcone reductase;****CHS***, *chalcone synthase;****COMT***, *caffeic acid 3-O-methyltransferase;****CSE***, *caffeoylshikimate esterase;****DFR***, *dihydroflavonol 4-reductase;****DFR***, *dihydroflavonol reductase;****F3’5’H***, *flavonoid 3’,5’-hydroxylase;****F3H***, *flavanone 3-hydroxylase;****FLS***, *flavonol synthase;****FNS***, *flavone synthase;****HCT***, *shikimate O-hydroxycinnamoyltransferase;****LAR***, *leucoanthocyanidin reductase;****PAL***, *phenylalanine ammonia lyase;****UFGT***, *UDP-glycose flavonoid glycosyltransferase*.

## LIST OF ABBREVIATIONS

Cas9: CRISPR-associated Protein 9
CHI: Chalcone Isomerase
CRISPR: Clustered, Regularly Interspaced Short Palindromic Repeat
DEGs: Differentially Expressed Genes
DSBs: Double-strand Breaks
H6H: Hyoscyamine 6β-hydroxylase
HDR: Homology-directed Repair
HMGS1: 3-hydroxy-3-methylglutaryl CoA synthase 1
HSS: Homospermidine Synthase
LLC: Lewis Lung Carcinoma
NAA: Naphthaleneacetic Acid
NGS: Next-genome Sequence
NHEJ: Nonhomologous End Joining
PCTOC: Plant Cell, Tissue and Organ Culture
TALENs: Transcription Activator-like Effector Nucleases
TF: Transcription Factors
UFGT: UDP-glycose Flavonoid Glycosyltransferase
ZFNs: Zinc-finger Nucleases
